# The Role of Supraerupted and Distoverted Maxillary Third Molars in the Treatment of Temporomandibular Disorder: A Randomised Controlled Trial

**DOI:** 10.7759/cureus.41158

**Published:** 2023-06-29

**Authors:** Narayanarao Gururaj, Ponniah Subramaniyan, Palraj Hasinidevi, Janani V

**Affiliations:** 1 Oral and Maxillofacial Pathology, CSI College of Dental Sciences and Research, Madurai, IND; 2 Oral and Maxillofacial Surgery, Dhanalakshmi Srinivasan Dental College, Perambalur, IND; 3 Dentistry, Srinivas Dental Clinic and Oral Care, Madurai, IND

**Keywords:** maxillary third molar, temporomandibular disorder, occlusal interferences, myofacial pain, clicking joint, headache

## Abstract

Objective

Temporomandibular disorder (TMD) is a multifactorial disease that is classified into muscular and joint disorders. The etiology of TMD is unknown but it is related to various factors such as bruxism, uncorrected high dental restorations, and occlusal prematurities. This study aims to provide treatment modalities for TMD patients with supraerupted and/or distoverted maxillary third molars that have premature contact with the opposing arch.

Methods

A total of 430 subjects diagnosed with TMD were included in the study and randomized into study and control groups based on their treatment needs. A detailed case history was taken, and findings of intra and extra oral examination were recorded along with other investigations such as study model analysis, orthopantomogram (OPG), cone-beam computed tomography (CBCT), and MRI. The multiphase treatment included counseling in phase I, extraction in phase II (only for the study group), and oral appliance in the third phase. The final phase involved the restoration of edentulous areas or reduced vertical dimension.

Results

Extraction of supraerupted and/or distoverted maxillary third molars in the study group during phase II showed a 96% reduction in TMD when compared to the control group who did not undergo extraction.

Conclusion

TMD is a repetitive motion disorder, and the success of treatment relies on the elimination of causative factors, the type of appliance used, and the establishment of ideal occlusion. This study suggests that the extraction of supraerupted and/or distoverted maxillary third molars is a prerequisite for treating TMD patients.

## Introduction

Temporomandibular disorders (TMD) are a group of disorders that cause pain in the jaw, head, neck, ears, and eyes, as well as deviation in the opening and closure of the mandible [1]. TMD is a significant public health issue that interferes with daily activities and significantly reduces the quality of life. The American Academy of Orofacial Pain [2] defines TMD as a collective term for several clinical problems that involve the muscles of mastication, the temporomandibular joint (TMJ), and its associated structures. Various factors, such as bruxism, clenching, stress, anxiety, occlusal disharmony, micro, and macro trauma, may be associated with TMD [3,4].

Numerous authors have discussed distinct classifications, etiological factors, clinical features, and treatment modalities for TMD [5,6]. The research diagnostic criteria (RDC) for temporomandibular disorders proposed a dual-axis system, consisting of axis I and II, for diagnosis based on signs and symptoms. The original RDC-TMD [7] was modified and recommended evidence-based new diagnostic criteria for temporomandibular disorders (DC-TMD) for both clinical and research settings. With the improvement of investigation protocols like orthopantomogram (OPG), CBCT, and MRI, it is now possible to distinguish between muscular disorders and those with pathological changes of the temporomandibular joint [8].

Psychosocial factors may also play a role in TMD [9], as suggested in a study. Alterations in head postures, spinal curves, and lower limbs were considered risk factors for muscular TMD [10]. While occlusion has been studied as a parameter in TMD patients and studied its possibility as a causative element, there is no direct correlation between occlusion and TMD [11].

A study suggested that mandibular functional shift is a type of malocclusion associated with crossbite, facial asymmetry, and TMJ pain [12]. It can be treated with a Michigan splint followed by orthodontic treatment. Another study [13] found a relationship between occlusion and TMD, with occlusion being one of the predisposing factors. The study observed a significant relationship between the distances of the center of the occlusal force, the asymmetry index of maximum occlusal force, and pain at the temporomandibular joint. A different study [14] recommended excluding the consideration of dental occlusion and pathophysiology of TMD as it is not always constantly hypothesized.

Scientific evidence suggests that the extraction of the third molar can have an effect on temporomandibular disorder (TMD) [4]. Signs and symptoms associated with TMD are common in patients referred for third molar extraction, but they may be due to pre-existing TMD and not related to the impacted tooth or its removal [15]. When an over-erupted tooth occupies a missing tooth area, it can cause occlusal interference and hinder smooth mandibular movements [16-18]. The over-erupted mandibular third molar in TMD patients has an impact on adjacent teeth located in the mandibular arch, causing them to incline mesially. However, removal of the over-erupted mandibular third molar alone is insufficient to correct chewing patterns, and it may be necessary to restore worn-out dentition. The association between the over-erupted tooth and electromyographic findings of muscles in both TMD and non-TMD individuals suggests a need for a clinical trial on the extraction of over-erupted teeth [19]. A study among 31 young adults with TMD found a significant association between premature contacts, occlusal stability, and TMD [20].

However, after conducting a thorough literature search, it is apparent that the presence of over-erupted, distally tilted maxillary third molars with missing or infra-occluded or impacted mandibular third molars in the opposing arch can cause premature occlusal contact, occlusal interference, occlusal disharmony, and prevent smooth mandibular movements. This can be an associated factor in causing or relieving TMD, but the evidence is inconclusive. Therefore, the aim of this study is to identify the presence of supra-erupted and/or distally tilted maxillary third molars in TMD patients with premature occlusal contact, occlusal interferences, and occlusal disharmony, and to provide a treatment regime with their removal as a prerequisite. The study also aims to identify the subjects with the presence of supra-erupted, distally tilted maxillary third molars having missing, infra-occluded, or impacted mandibular third molars in the opposing arch, to consider the supra-erupted, distally tilted maxillary third molar as a factor associated with TMD, and to adopt a management protocol by extraction of supra-erupted and/or distally tilted third molars as a prerequisite in reducing symptoms, especially neck ache, headache, and clicking joint.

## Materials and methods

This randomized controlled trial with a concurrent parallel design was conducted among 430 purposively selected patients between the years 2017 and 2022 at a private dental clinic in Madurai, Tamil Nadu, India. The subjects were aged 18 years and above and reported with any of the following symptoms: headache, neck pain, pre-auricular pain, dull facial pain, and clicking noise heard at the temporomandibular joint (TMJ). All the patients were diagnosed with TMD by a single examiner based on the Diagnostic Criteria for Temporomandibular Disorders (DC-TMD). Out of 430 patients, 206 patients were diagnosed with arthrogenous TMD and remaining 224 patients were diagnosed with muscular TMD. Patients suffering from dental pain due to pulpal or periodontal origin or with any known systemic diseases such as osteoarthritis, cervical spondylitis, eye problems, otolaryngology problems, and migraine-related headaches were excluded from the study.

**Figure 1:**
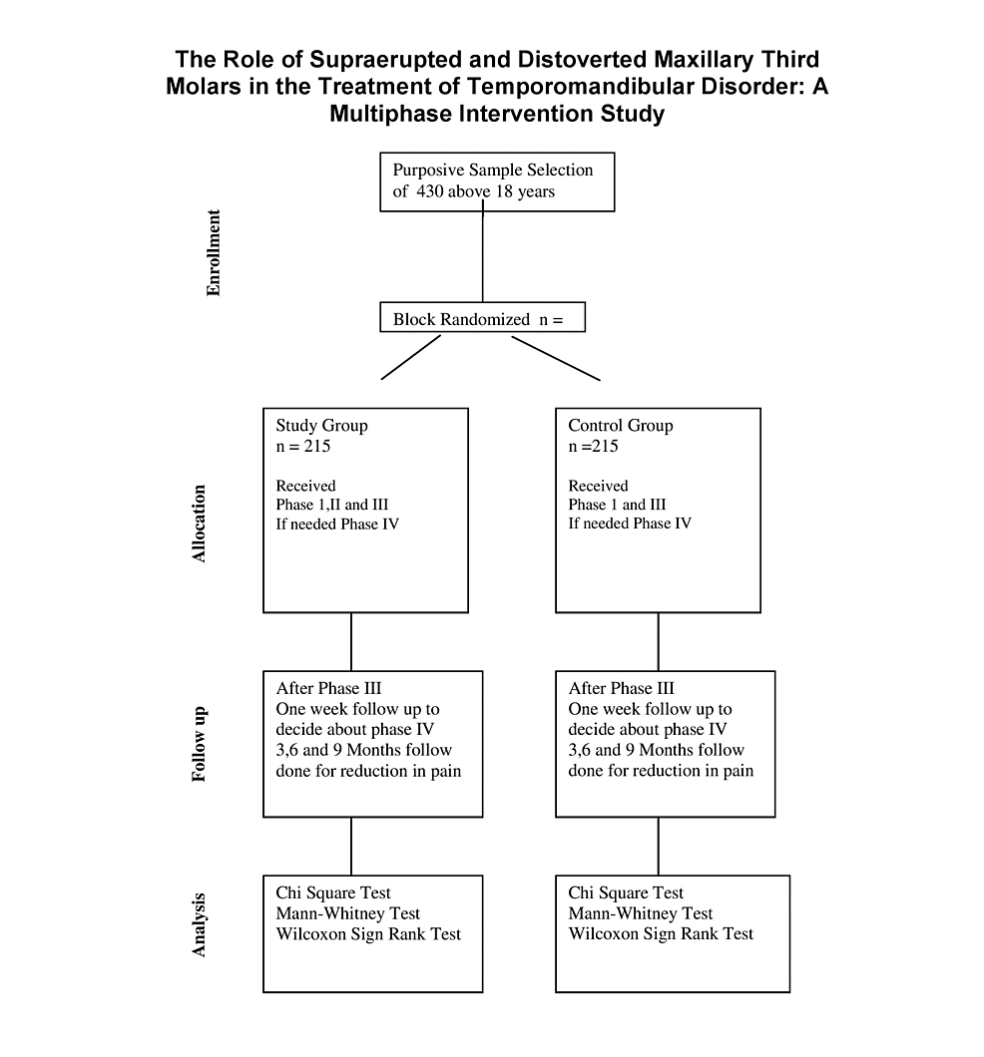
Consort Diagram

The selected study subjects were divided into a study group (215 patients) and a control group (215 patients) using the block randomization method and participant flow throughout the study is depicted in the CONSORT flowchart (Figure 1). The flowchart illustrates the progression of participants from enrollment to the completion of the study without any dropouts due to non-compliance or intolerance to treatment.

The occlusal registration was carried out using 8 microns thickness articulating paper (Bausch, Germany) and bite registration wax (Alu Wax, India). The occlusal status and premature contacts of all patients were assessed by studying model analysis [21]. Orthopantomography was advised for all patients for initial screening, and cone-beam computed tomography (CBCT NEWTOM GO, Italy) was performed in selected patients to study the distally tilted maxillary third molars and changes in the condyle of the mandible or eminence. Magnetic resonance imaging (MRI) was advised to study the articular disc in selected patients. To determine pain perception, the Visual Analogue Pain Rating Scale was used in both the study and control groups at the end of three, six, and nine months.

The patients underwent a multiphase treatment approach and the duration of phases was decided based on subjective and objective disappearance of the symptoms. In the first phase, counseling was prioritized. The second phase involved the extraction of distally tilted and/or supra-erupted maxillary third molars. In the third phase, custom-fabricated oral appliances, such as anterior repositioning appliances, stabilization appliances, and/or deprogramming appliances, were provided based on each patient's needs. Once the patients' symptoms had subsided, the fourth phase was initiated.

Based on individual patient needs, the fourth phase of treatment included procedures such as the removal of impacted teeth, orthodontic correction, restoration of partially edentulous areas, and correction of vertical dimension, among others [22]. In both the study and control groups, patients underwent counseling during phase I treatment, but only the study group proceeded to phase II (extraction of distally tilted and/or supra-erupted maxillary third molars) one week after phase I. After phase II, the study group patients entered phase III treatment, while the control group patients proceeded directly to phase III one week after phase I. Follow-up was conducted for both groups, and phase IV treatment was provided as necessary. The study was conducted with institutional ethical clearance from CSI College of Dental Sciences and Research in Madurai, and all patients provided written informed consent to participate.

## Results

Statistical analysis was performed by entering data into Microsoft Excel for Windows (Microsoft® Corp., Redmond, WA, USA) and frequency distribution and percentages were calculated using SPSS version 17 (SPSS Inc., Chicago, IL, USA) for Windows. Chi-Square test, Mann-Whitney Test, and Wilcoxon sign rank test were used to identify significant differences between the two groups, with P<0.05 considered statistically significant.

The study enrolled a total of 430 patients, with 215 in the study group and 215 in the control group. All participants completed the entire study without any dropouts due to non-compliance or intolerance to either treatment. The demographic distribution showed that approximately 60% (n=256) of the patients were female, and the remaining 40% (n=174) were male. The mean age of the study subjects was 38.45±11.92 years. Table 1 provides information on the distribution of patients based on their clinical features, dental status, and past medical history.

**Table 1:** Signs and symptoms, intraoral findings, past medical history, and habits

Clinical data	Study group (215)	Control group (215)
Mean age	37.85 ± 12.08	38.95 ± 11.72
Sex	Male – 109, Female – 106	Male – 106, Female – 109
Acute severe pain at TMJ region upon movement of jaws	32	29
Headache	156	144
Neck pain	88	84
Reduced mouth opening	98	92
Clicking joint	104	102
Distally titled and over-erupted maxillary third molars	38	40
Supraerupted maxillary third molar	42	52
Generalized attrition and loss of vertical dimension	44	24
Partially edentulous areas with tilted teeth	12	34
Bruxism	164	92
Occupational stress	184	112

The outcomes of TMD treatments were assessed and are shown in Table 2. The extraction was performed on patients only in the study group having supraerupted and/or distoverted maxillary third molars. To determine the contribution of extraction versus the prolonged duration of phase I treatment in alleviating pain, we compared the pain outcomes between the study group (undergoing extraction) and a control group that received the same duration of phase I treatment but did not undergo extraction. By examining the pain relief in both groups, we aimed to evaluate the specific impact of extraction on pain reduction. The study group exhibited significantly greater pain relief compared to the control group, it would suggest that the extraction procedure played a role in alleviating pain.

**Table 2:** Various phases of treatment NSAIDs: Non-steroidal anti-inflammatory drugs

Phase I	Phase II	Phase III	Phase IV
Counselling. 1. To avoid wide opening of the mouth while eating or yawning. 2. To avoid eating hard candy or any hard food items. 3. To apply hot fomentation over the face. 4. To take soft diet. 5. To sit or stand in the proper position without bending for a long time. 6. Not to hold a mobile phone in the wrong position. 7. To sleep regularly on time to reduce anxiety and stress. 8. Eat or chew on both sides of the jaws. 9. NSAIDS for pain.	Extractions of distally tilted and or supra-erupted maxillary third molars	Intraoral appliances	Replacement of missing tooth and restoration of partially edentulous areas. Extraction of impacted teeth. Orthodontic correction. Restoration of loss of vertical dimension.

Post-extraction, a complete disappearance of symptoms such as headache, neck pain, clicking sound, acute severe pain at the TMJ region upon jaw movement, and muscle tenderness was observed in 92% of patients during the 60-day postoperative follow-up period (as shown in Table 3). Subsequently, the third phase of treatment was rendered.

**Table 3:** Immediate follow-up for phase II and phase III

Complete disappearance of symptoms following phase II		Complete disappearance of symptoms following phase III		
Duration	Extraction of distoverted, over-erupted maxillary third molars in 60 (N)	Duration	Study group (215)	Control group (215)
15 days	43 (72%)	60 Days	154 (72%)	52 (24%)
30 days	48 (80%)	180 Days	189 (88%)	90 (42%)
60 days	55 (92%)	270 Days	206 (96%)	112 (52%)

The Visual Analogue Scale pain rating was utilized to assess the pain perception of the subjects experiencing acute severe pain upon jaw movement, both in the study and control groups, at baseline, three months, six months, and nine months, to determine their pain status before and after treatment. The pain rating between the study and control groups at baseline was found to be similar, with 15 and 16 subjects reporting very severe pain, and 12 and nine subjects reporting severe pain, respectively. However, at the end of nine months, there was a statistically significant reduction in the pain rating among the study group, with 25 subjects reporting no pain and four reporting mild pain. In contrast, in the control group, all 29 subjects reported moderate to the worst pain (Table 4).

**Table 4:** Pain rating scale in baseline, three, six and nine months between study and control group

Duration (Study Group)	No Pain	Mild	Moderate	Severe	Very Severe	Worst Pain	Total
Baseline	0	0	0	12	15	5	32
3 months	0	0	15	12	3	2	32
6 months	0	13	15	3	1	0	32
9 months	25	4	2	1	0	0	32
Duration (Control Group)	No Pain	Mild	Moderate	Severe	Very Severe	Worst Pain	Total
Baseline	0	0	0	9	16	4	29
3 months	0	0	0	9	16	4	29
6 months	0	0	15	6	5	3	29
9 months	0	0	18	7	2	2	29

In the Chi-square test, it was found that the disappearance of symptoms in the study population through the extraction of supraerupted and distoverted teeth was statistically significant with a p-value of less than .000 at the end of two months (as shown in Table 3).

For the control group patients who directly entered phase III treatment one week following phase I, a bruxism guard was given to 34 patients, an anterior repositioning appliance for 90 patients, and a stabilization appliance for 16 patients.

**Figure 2:**
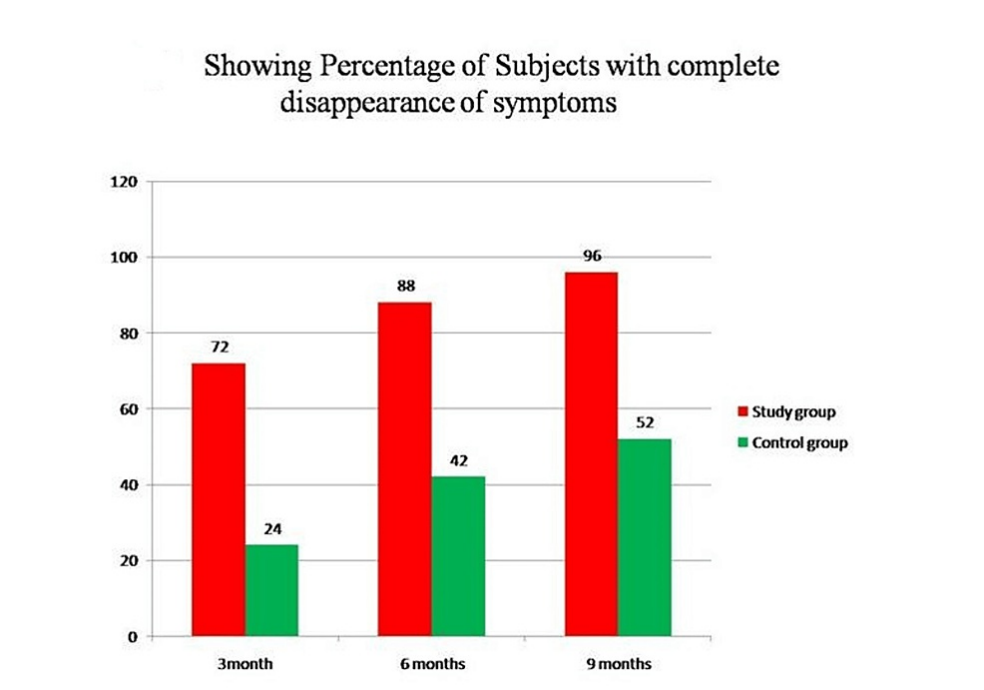
Percentage of symptoms with complete disappearance of symptoms

The control group showed a 52% complete disappearance of symptoms at the end of nine months. However, 54% of these patients experienced a recurrence of symptoms by the end of 12 months. In comparison, the study group had a 96% complete disappearance of symptoms at the end of nine months, and none of the study subjects had a recurrence of symptoms by the end of the nine-month follow-up period. Therefore, when considering the overall follow-up period of nine months, the study group showed better results than the control group (as shown in Figure 2 and Table 3).

The Chi-square test, Mann-Whitney test, and Wilcoxon sign rank test revealed that there is statistical significance between the study group and control group at three, six, and nine months, with a p-value of 0.000.

## Discussion

Temporomandibular disorder (TMD) is a complex condition with a multifactorial etiology and poorly understood pathogenesis [23]. The exact causes of TMD are still debatable, and eliminating these factors can be perplexing for clinicians trying to find the best treatment. Treatment for TMD is complicated, and requires a treatment protocol that can reduce chronic pain and restore joint stability. Various treatment options have been advocated, including clinical management, physiotherapy, oral appliances, and injections in joints and muscles. However, there is no one specific treatment protocol that is universally applicable to all types of TMD.

In our study, we found that patients were often not referred to dental clinics initially, but were instead identified and treated by ophthalmologists, neurosurgeons, orthopaedic surgeons, and otolaryngology surgeons based on their symptoms. Some patients with acute severe pain were clinically diagnosed with trigeminal neuralgia and treated by neurologists before being referred to a dentist. In our study, muscular TMD was more common, with headaches being the main complaint followed by joint TMD with internal derangement (clicking joint) [1].

**Figure 3:**
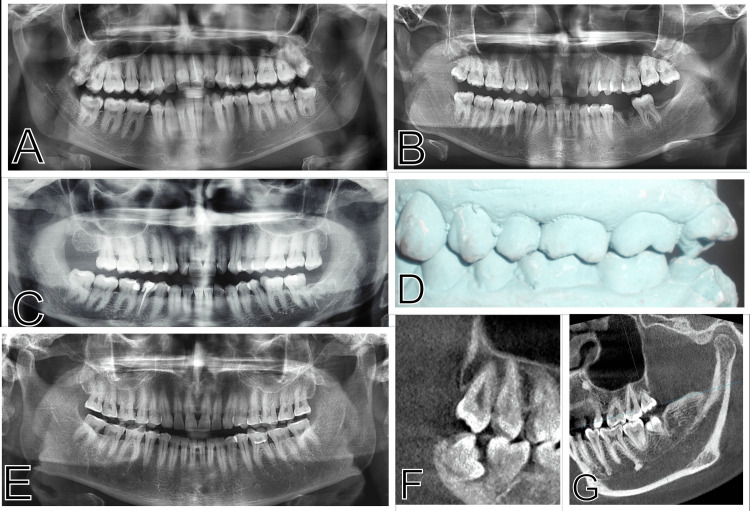
Orthopantomograph, cone beam CT, plaster study model (A) Tilted maxillary third molars in orthopantomograph. (B) Tilted maxillary third molars with supraerupted left maxillary third molar having missing tooth in the lower arch in orthopantomograph. (C) Supraerupted left maxillary third molar having missing tooth in the lower arch in orthopantomograph. (D) Articulated plaster cast with tilted left maxillary third molar with palatal cusp contacting lower tooth. (E) Severe attrition of teeth in orthopantomograph. (F) Distoverted right maxillary third molar in CBCT. (G) Condyle morphology in CBCT.

Imaging played a major role in diagnosis and treatment planning [24]. From the panoramic radiographs (Figure 3A-3C), we observed the tilt of maxillary third molars and supraeruption. In our study, premature contacts and occlusal interferences were assessed using imaging as well as articulated casts (Figure 3D, 3E). Cone beam computed tomography (CBCT) revealed the supraerupted, distoverted aspects of a maxillary third molar with condylar morphology (Figure 3F, 3G).

Numerous authors have recommended a precise treatment protocol that reduces the load on the temporomandibular joint and muscles to achieve the goal of treating TMD patients [2]. The primary objective of the treatment is to eliminate occlusal interferences, chronic myofascial pain, neck pain, head pain, and joint discomfort caused by various factors and to reduce the psychological burden [25]. Occlusal splints or oral appliances are given to manage both muscular and joint disorders, along with medical management [26,27].

In this present study, a multiphase treatment regimen was provided for the study group. Following counseling in phase I, patients with distally tilted or supra-erupted maxillary third molars having premature contacts with the opposing arch were considered for extraction in phase II of treatment. Extraction of the distoverted maxillary third molars in the study group showed a drastic improvement, and 96% of the study subjects achieved good mouth opening, disappearance of clicking, headache, neck pain, preauricular pain, and pain in and around the orbit on the 60th day. About 96% of the study group patients showed complete disappearance of symptoms following phase I and II within two months. The absence of symptoms stated by the patients following the extraction of distally tilted maxillary third molars indicates the disappearance of premature contact and restores the smooth mandibular movements. Immediately after phase II, the patients in the study group received phase III treatment.

Patients in the control group entered phase III treatment directly after phase I, while patients in the study group entered phase III following phase II. A custom-fabricated intraoral appliance made of hard acrylic was delivered to the patients in both groups as the third phase of the treatment [28]. The selection of the appliance, whether it's an anterior repositioning appliance, stabilization appliance, or a muscle deprogrammer, is based on the type of disorder, whether it's muscular, joint, or a combination of both. It was informed to the patients that the appliances are a temporary remedy and not a permanent one, and the final treatment would be the correction of problems like restoration of partially edentulous areas, reduced vertical dimension, and malocclusion at the final stage of the treatment.

For patients with severe bruxism, a bruxism guard [29,30], or splint (deprogrammer) made of hard acrylic was given for a period until the habits disappear. An occlusal guard greatly reduces the stress on masticatory muscles and redistributes the forces equally over the jaw. For patients with a clicking joint, an anterior repositioning appliance made of hard acrylic is provided and recommended to wear overnight [28,29].

In this present study, for patients with acute severe pain that worsens upon any feasible movements of jaws with severe attrition and loss of cuspal facets a stabilization splint made of hard acrylic was delivered that reduced the symptoms gradually. The stabilization splint rests the joint completely from movement and reduces the inflammation at the retrodiscal area and joint interface by preventing frequent friction [30].

Once the signs and symptoms had completely alleviated, the fourth phase of treatment was carried out for both the study and control groups as required. Follow-up observations showed that the study group, which received phase II treatment followed by phase III, had better results than the control group (as shown in Table 3). The visual analog pain rating scale revealed a considerable reduction in pain perception among the study group compared to the control group, indicating that the treatment modalities followed in the study group were found to be highly effective in reducing symptoms in TMD patients. Therefore, the extraction of supraerupted or distally tilted maxillary third molars should be considered a prerequisite when planning treatment for TMD patients.

One limitation of this study is that we did not measure the degree of inclination and over-eruption of the maxillary third molar, which could have been a factor. Another limitation was the challenge of gaining patients' trust, as they may have received conflicting opinions from other specialists. The study also revealed that it was difficult to educate patients about the need and purpose of the custom-made hard acrylic appliance, as opposed to ready-made soft splints. Additionally, patients had difficulty understanding the concept of TMD and the various treatment aspects, which varied from patient to patient and resulted in delayed outcomes.

## Conclusions

This study highlights the significant benefits of extracting supraerupted and/or distally tilted maxillary third molars in reducing headache and neck pain in patients with TMD. Thus, the extraction of such teeth should be considered a necessary step in the treatment of TMD patients, in addition to other treatment protocols. Our findings suggest that identifying supraerupted, distally inclined maxillary third molars with premature contact with the opposing arch is a contributing factor in TMD and removing these teeth greatly improves successful management, reduces patient burden, and minimizes recurrence. However, this study has certain limitations, such as not considering the degree of inclination and over-eruption of the maxillary third molar, difficulty in getting patients' confidence, and educating them on the need and purpose of custom-made hard acrylic appliances. Future large-scale studies are needed to validate our findings.

## Figures and Tables

**Figure 4:**
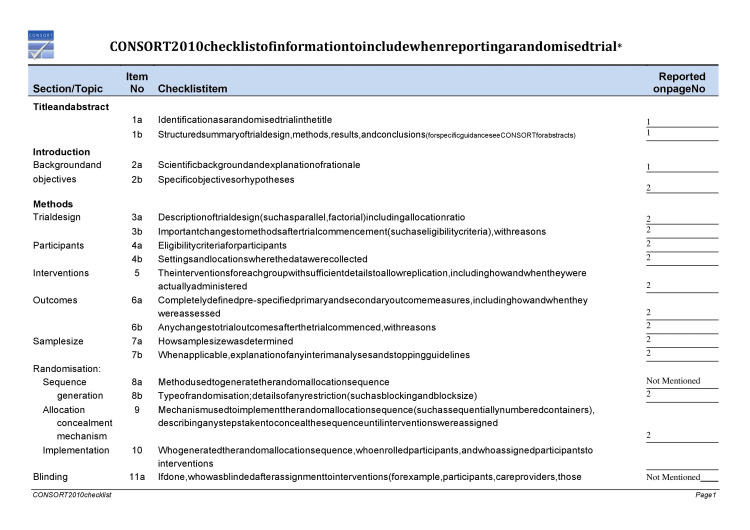
Consort checklist page 1

**Figure 5:**
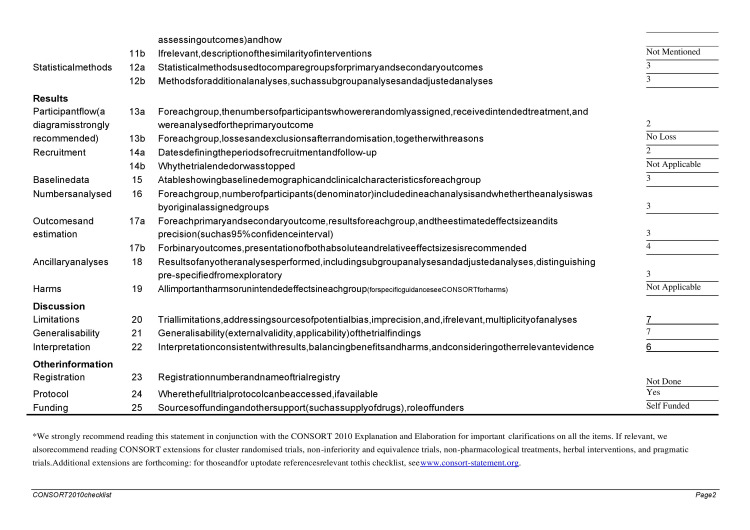
Consort checklist page 2
